# In Silico Lead Identification of *Staphylococcus aureus* LtaS Inhibitors: A High-Throughput Computational Pipeline Towards Prototype Development

**DOI:** 10.3390/ijms262412038

**Published:** 2025-12-14

**Authors:** Abdulaziz H. Al Khzem, Tagyedeen H. Shoaib, Rua M. Mukhtar, Mansour S. Alturki, Mohamed S. Gomaa, Dania Hussein, Ahmed Mostafa, Layla A. Alrumaihi, Fatimah A. Alansari, Maisem Laabei

**Affiliations:** 1 Department of Pharmaceutical Chemistry, College of Pharmacy, Imam Abdulrahman Bin Faisal University, P.O. Box 1982, Dammam 31441, Saudi Arabia; msalturki@iau.edu.sa (M.S.A.); msmmansour@iau.edu.sa (M.S.G.); ammostafa@iau.edu.sa (A.M.); 2 Department of Pharmaceutical Chemistry, Faculty of Pharmacy, University of Gezira, Wad Madani 21111, Sudan; shoaibth37@hotmail.com (T.H.S.); ruamubarak1@gmail.com (R.M.M.); 3 Department of Pharmacology, College of Pharmacy, Imam Abdulrahman Bin Faisal University, Khobar 31441, Saudi Arabia; dahussein@iau.edu.sa; 4 College of Pharmacy, Imam Abdulrahman Bin Faisal University, P.O. Box 1982, Dammam 31441, Saudi Arabia; 2210000333@iau.edu.sa (L.A.A.); 2220001110@iau.edu.sa (F.A.A.); 5 School of Cellular and Molecular Medicine, University of Bristol, Bristol BS8 1TD, UK

**Keywords:** multi-drug resistance, *Staphylococcus aureus*, lipoteichoic acid, high-throughput screening, natural products, lead identification, drug design and discovery

## Abstract

The emergence of multidrug-resistant *Staphylococcus aureus* underscores the urgent need for novel therapeutic agents targeting essential bacterial pathways. The lipoteichoic acid synthase (LtaS) is crucial for the synthesis of lipoteichoic acid in the cell wall of Gram-positive bacteria and represents a promising and vulnerable target for antimicrobial drug development. This study employed a comprehensive computational pipeline to identify potent inhibitors of the LtaS enzyme. A library of natural compounds was retrieved from the COCONUT database and screened against the crystal structure of the extracellular domain of LtaS (eLtaS) (PDB ID: 2W5R, obtained from the Protein Data Bank) through a multi-stage molecular docking strategy. This process started with High-Throughput Virtual Screening (HTVS), followed by Standard Precision (SP) docking, and culminated in Extra Precision (XP) docking to refine the selection of hits. The top-ranking compounds from XP docking were subsequently subjected to MM-GBSA binding free energy calculations for further filtration. The stability and dynamic behavior of the resulting candidate complexes were then evaluated using 100 ns molecular dynamics (MD) simulations, which confirmed the structural integrity and binding stability of the ligands. Density Functional Theory calculations revealed that screened ligands exhibit improved electronic stabilization and charge-transfer characteristics compared to a reference compound, suggesting enhanced reactivity and stability relevant for hit identification. Finally, ADMET (Absorption, Distribution, Metabolism, Excretion, and Toxicity) profiling was conducted to assess the drug-likeness and pharmacokinetic safety of the lead compounds. These findings support them as promising orally active leads for further optimization. Our integrated approach shortlisted eight initial hits (A–H) that showed interesting scaffold diversity and finally identified two compounds, herein referred to as Compound **A** and Compound **B**, which demonstrated stable binding, favorable free energy, and an acceptable Absorption, Distribution, Metabolism, and Excretion, and Toxicity (ADMET) profile. These candidates emerge as promising starting points for developing novel anti-staphylococcal agents targeting the LtaS enzyme that cand be further proved by experimental validation.

## 1. Introduction

*Staphylococcus aureus* is a versatile and opportunistic Gram-positive bacterium that causes a wide spectrum of infections, ranging from superficial skin and soft tissue infections to life-threatening conditions such as pneumonia, osteomyelitis, endocarditis, and sepsis [1,2,3]. Of particular clinical concern is methicillin-resistant *Staphylococcus aureus* (MRSA), which has become a global health threat due to its resistance to multiple β-lactam antibiotics and other commonly used antimicrobials [4]. MRSA infections are associated with increased morbidity, mortality, and healthcare burden worldwide. Alarmingly, MRSA has been estimated to cause more annual deaths in the United States than the combined toll of HIV, tuberculosis, and viral hepatitis [5,6]. In response to this growing crisis, the World Health Organization (WHO) has designated MRSA as a high-priority pathogen, urging the development of new antibacterial strategies [7].

**Figure 1:**
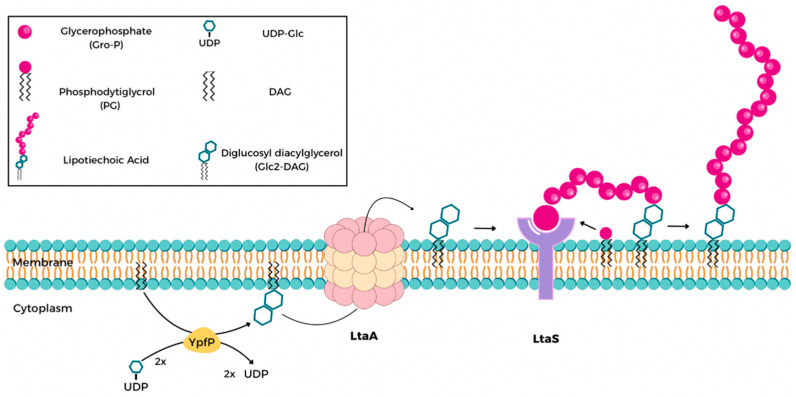
Overview of the lipoteichoic acid (LTA) biosynthetic pathway in *S. aureus*. Lipoteichoic acid (LTA) biogenesis begins with the creation of the diglucosyl-diacylglycerol (Glc_2_-DAG) anchor. The enzyme diacylglycerol β-glucosyltransferase (YpfP) catalyzes the reaction between diacylglycerol (DAG) and UDP-glucose to generate this membrane anchor. Next, the glycolipid permease LtaA transports the Glc_2_-DAG molecule from the inner to the outer leaflet of the cytoplasmic membrane. Finally, the enzyme LtaS cleaves the glycerolphosphate headgroup from phosphatidylglycerol (PG) and sequentially transfers approximately 15 to 50 glycerolphosphate units to the Glc_2_-DAG anchor.

Traditional treatment options for MRSA infections rely heavily on glycopeptides, oxazolidinones, and lipopeptides. However, the emergence of resistance to these last-resort antibiotics highlights the urgent need to explore novel targets and mechanisms of action [8,9,10,11,12]. One such promising target is the lipoteichoic acid synthase (LtaS), a key enzyme responsible for the synthesis of lipoteichoic acids (LTAs), which are anionic glycopolymers embedded in the cell membranes of Gram-positive bacteria, see Figure 1 [13,14,15]. LTAs play essential roles in bacterial physiology, including cell wall integrity, ion homeostasis, autolysin regulation, and pathogenesis [16,17,18]. Genetic or pharmacological disruption of LtaS has been shown to impair bacterial growth, induce morphological defects, and increase susceptibility to host immune factors and antibiotics [17,18,19,20,21]. Importantly, as LtaS is conserved among Gram-positive organisms but absent in humans, it offers an attractive therapeutic window for selective inhibition [18].

Progress in research and the development of novel antibiotics with distinct mechanisms of action, coupled with high efficacy and low toxicity, are essential to minimizing the risk of drug resistance and broadening the range of available antimicrobial agents [22]. In this context, LtaS emerges as a promising yet underexplored antibacterial target. To date, only a few small-molecule inhibitors have been reported against LtaS, most notably, the dye Congo red, which is limited by toxicity concerns [17]. Another compound, 1771, has been reported to reduce LTA levels and was initially suggested as an LtaS inhibitor. However, more recent studies have cast doubt on 1771’s direct inhibition of LtaS, indicating that it likely exerts its effects through an alternative mechanism and that its precise molecular target remains unclear [12,17,19]. Given this lack of pharmacological advancement, further investigation into LtaS holds significant potential for identifying new antimicrobial agents and deepening our understanding of bacterial physiology.

Natural products (NPs) have historically played an essential role in the development of antibiotics, serving as the foundation for many clinically approved antimicrobial agents. Their structural diversity and evolutionary refinement for biological activity make them a rich source of pharmacologically relevant molecules [23,24]. However, the sheer number and complexity of available natural compounds necessitate efficient strategies for identifying promising candidates. In this context, virtual screening (VS) and molecular modeling approaches, such as molecular docking, Molecular Mechanics-Generalized Born Surface Area (MM-GBSA) binding energy calculations, and Molecular Dynamics (MD) simulations, offer cost and time-effective strategies to identify and optimize novel ligands against challenging targets like LtaS [25,26,27,28,29,30]. In this study, we employed a comprehensive computational pipeline comprising molecular docking, MM-GBSA calculations, ADMET (Absorption, Distribution, Metabolism, and Excretion, and Toxicity) profiling, and MD simulation to filter NPs of the COCONUT library, aiming to identify novel LtaS inhibitors with potential antibacterial activity against MRSA.

## 2. Results and Discussion

### 2.1. Molecular Docking

Molecular docking is a widely employed computational technique in drug discovery that predicts the preferred orientation of small molecules when bound to a target protein, estimating their binding affinity and stability within the active site. This method enables the rapid screening of large compound libraries to identify potential inhibitors by simulating ligand–protein interactions and ranking compounds based on scoring functions. These scoring functions evaluate various intermolecular forces such as hydrogen bonding, hydrophobic interactions, and electrostatic attractions to estimate how well a ligand fits into the binding site [27,28,29,30]. Docking thus serves as an essential initial step to prioritize candidates for further detailed analysis, accelerating the drug development process. To ensure the reliability of the docking approach employed in this study, we first validated our protocol before proceeding with large-scale screening.

To confirm the accuracy of the docking protocol, a validation step was performed by re-docking the co-crystallized ligand into the active site of eLtaS using the same parameters intended for virtual screening. The resulting docked pose was then compared to the original crystallographic conformation by calculating the RMSD between the two structures. An RMSD of 1.1742 Å was obtained, indicating a high degree of structural overlap and confirming that the docking method could accurately reproduce the experimentally observed binding mode. In addition, the docking score of the natural substrate was found to be −10.062 kcal/mol, supporting the validity of the scoring function within this system.

Following validation, we implemented a tiered docking strategy to virtually screen the compound library against eLtaS. The virtual screening campaign was conducted using the COCONUT natural product database which contains 695,133 compounds. A filtration protocol based on a set of calculated physicochemical descriptors and pharmacokinetic parameters was applied and resulted in a total of 40,332 compounds for virtual screening. Our main purpose when designing our filtration criteria was to retain compounds with high potential for preclinical development and clinical translation especially in the absence of a crystal structure of a bound inhibitor. In brief, the database was downloaded and filtered using Schrödinger’s Canva to remove duplicates and apply certain filtration criteria, including MW 100–600, logP −3–6, number of rings < 6, total heavy atoms count < 50, total charge −4–4, number of H-donors < 7, number of H acceptors < 12, number of rotatable bonds < 11, and total charge −4–4. Additionally, a natural product-like score < 2 was used. Quantitative estimation of drug likeness (QED) was the final criteria to finetune the filtration process as it integrates eight properties: molecular weight, LogP, H-bond donors, H-bond acceptors, charge, aromaticity, stereochemistry, and solubility, with a score from 0–1. A higher score means the molecule is more drug-like. The QED filtration score was set to >0.2. virtual databases. This protocol if proven successful would streamline future database preparation across other virtual databases for virtual screening to develop experimental lead for Ltas. All ligands were preprocessed and energy-minimized using the MacroModel tool to ensure proper geometry and conformational flexibility. The prepared library was docked against eLtaS using the Glide module within the Schrödinger suite. An initial screening was performed in HTVS mode, reducing the number of candidates to 12,376 compounds. These were then subjected to a sequential docking protocol, progressing through SP and finally XP docking modes to refine the selection based on predicted binding affinities and interaction profiles. The final set exhibited variable docking scores. Hence, to enhance the reliability of hit selection, all compounds with docking scores equal to or better than −6.0 kcal/mol (a total of 268 compounds) were advanced for MM-GBSA binding free energy calculations, allowing for more rigorous post-docking evaluation. This threshold was chosen to retain a diverse set of moderately scoring compounds, enabling a broader chemical space to be reassessed using a more accurate free energy estimation method.

### 2.2. MM-GBSA Calculations

MM-GBSA is a post-docking free energy estimation method that offers a more rigorous assessment of ligand–protein binding affinities. Unlike docking scores, which are based on simplified scoring functions, MM-GBSA incorporates molecular mechanics energies, solvation effects, and surface area terms to estimate the binding free energy (ΔG_bind) of ligand–receptor complexes [31]. This approach provides a more subtle and physically realistic evaluation of binding strength, making it particularly useful for refining virtual screening results and prioritizing top candidates for further study. Accordingly, MM-GBSA was applied to the top-ranked compounds from docking to enhance the reliability of hit selection.

A total of 268 compounds, filtered from the docking stage based on docking scores ≤ −6.0 kcal/mol, were subjected to MM-GBSA binding free energy calculations to refine hit selection. These calculations were performed using the Prime MM-GBSA module to estimate the ΔG_bind values of each ligand–eLtaS complex. The MM-GBSA method provided a more rigorous assessment of binding affinity by incorporating molecular mechanics energies and solvation effects.

To benchmark the results, phodphatidylglycerol the natural substrate of LtaS (used as a reference) was included in the MM-GBSA calculations and yielded a binding free energy of −30.15 kcal/mol.

A combined approach was used to shortlist hit compounds based on both docking scores and calculated binding affinities since no strong correlation is established in the literature between either calculation methods with experimental inhibition values. The selection was based on two criteria: the first one is having an MM-GBSA ≥ that of the reference ligand and the second one is having a docking score ≥ that of the reference ligand subject to also showing a favorable binding energy (MM-GBSA below zero). This led to the identification of eight primary hits (A–H).

Final hits were restricted to compounds with ΔG_bind values more favorable than the reference. Notably, only two compounds surpassed this threshold: Compound **A** (ΔG_bind = −46.02 kcal/mol), Compound **B** (ΔG_bind = −32.78 kcal/mol). Those two hits were further promoted to MD simulations and DFT calculations to substantiate their binding stability and efficiency. The docking scores and ΔG_bind energies of the top compounds and the natural substrate are presented in Table 1.

Although the natural substrate exhibited a superior docking score (−10.062 kcal/mol) compared to the selected hits (which scored in the −6.0 kcal/mol range), its MM-GBSA binding free energy was comparatively less favorable. This observation underscores the added value of MM-GBSA as a post-docking refinement method, capable of capturing energetic effects, such as solvation and desolvation, that are not fully accounted for in docking algorithms. As such, MM-GBSA provides a more comprehensive view of ligand–protein binding stability and supports the re-ranking of candidates based on thermodynamic favorability. While the MM-GBSA results indicate a favorable binding affinity for the selected compounds, further computational evaluation is necessary to prioritize these compounds for preclinical testing based on their calculated scores. Moreover, A solid conclusion has been stated in the provided reference [31]. In particular, the methods of MM-GBSA may be useful to improve the results of docking and virtual screening or to understand observed affinities and trends. However, they are not accurate enough for later states of predictive drug design. Hence, we have chosen compound **A** and compound **B** for additional analysis using MD simulations and DFT calculations to validate their superior MM-GBSA scores.

The initial hit list showed an interesting pharmacophore structure composed of two aromatic rings bearing an acidic functionality on both rings, mainly a phenolic and a phosphate group with a flexible spacer that accommodated from 4 (hit C) to 10 (hit B) atoms. The spacer contains heteroatoms capable of H bond formation. Scaffold diversity was also represented in hit B where one of the aromatics bearing a CN group instead of an acidic functionality and in hit E where an aliphatic acyclic scaffold bearing acidic functionality on only one side of the chain.

### 2.3. Ligand–Protein Interactions Analysis

**Figure 2:**
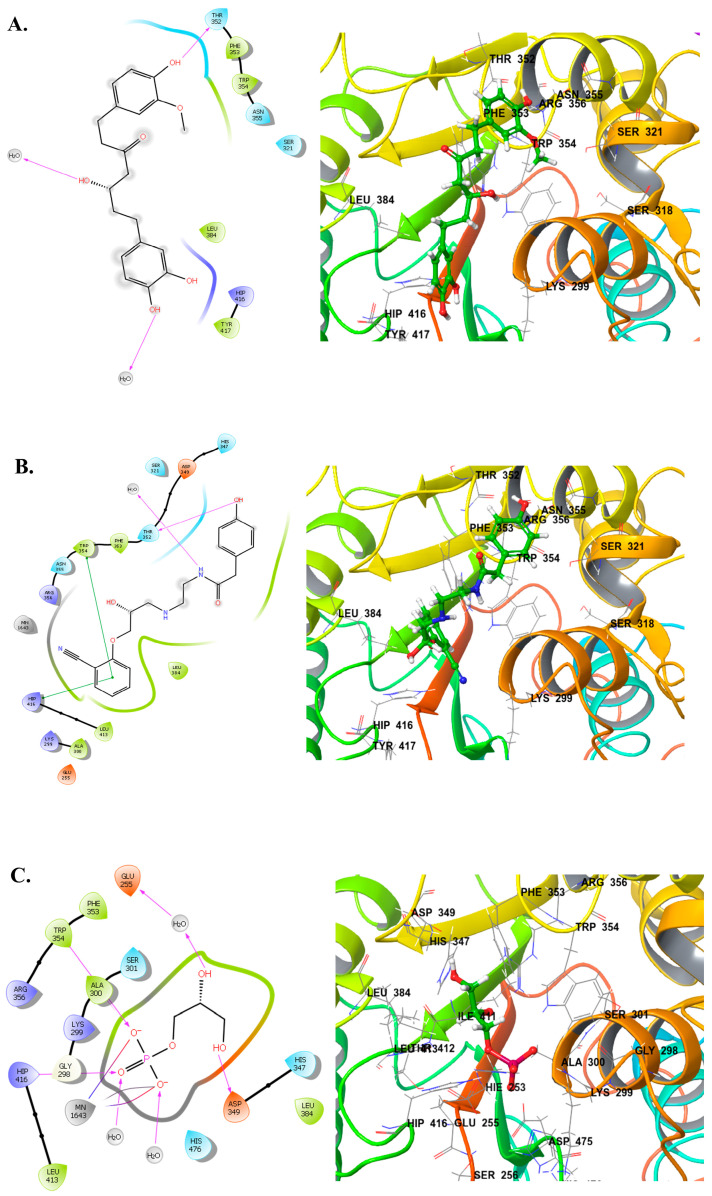
Binding interactions of top hit compounds with the target protein active site. (**A**–**C**) The 2D and 3D interaction diagrams illustrating the binding poses of the three top-ranked compounds within the enzyme active site. Key interacting residues forming hydrogen bonds, hydrophobic contacts, π–π stacking, and water-mediated interactions are highlighted. Residues such as Phe353, Trp354, Thr352, Arg356, and Lys299 are prominently involved in stabilizing ligand binding. Water molecules and coordinated metal ion interactions contributing to complex stability are indicated where relevant. The ligand binding conformations demonstrate strong complementarity to the active site pocket, supporting their potential as promising inhibitors.

To further understand the binding behavior of the top candidates identified through MM-GBSA (Compound **A** and **B**), a detailed analysis of their binding modes within the LtaS active site was conducted. These compounds exhibited interactions with residues critical for enzymatic function and shared several binding features with the natural substrate. The 2D and 3D interaction diagrams of the top compounds and the reference are presented in Figure 2.

Compound **A** formed a hydrogen bond with THR352 and established hydrophobic interactions with TYR417 H2O2002, and 2435

Compound **B** demonstrated the most diverse interaction profile. It formed a hydrogen bond with THR352, π–π stacking with HIP416 and TRP354, and polar contact with H_2_O2002.

The natural substrate, used as a reference, formed hydrogen bonds with GLU255, ASP349, TRP354, and HIP416, hydrophobic contacts with ALA300, PHE353, TRP354, LEU384, and LEU413. It also involved in charged positive interactions with LYS299, ARG356, and HIP416, and charged negative interactions with GLU255 and ASP349. Polar contacts with SER301, HIS347, and HIS476 were made. Also, two salt bridges and a metal coordination with Mn^2+^ ion, engaging the metal center critical for catalysis were observed.

When mapped against these structurally validated residues, all compounds showed substantial overlap: Compound **A** interacts with TRP354, PHE353, LEU384, and HIP416—all associated with substrate binding or stabilization. Compound **B** demonstrates the broadest engagement, contacting not only substrate-binding residues like HIS347, ASP349, PHE353, TRY354, ARG356, and LEU384, but also metal coordination residues such as GLU255 and ASP349.

These consistent interactions, particularly with functionally essential residues, provide compelling evidence that all top compounds bind within the core catalytic site of eLtaS, positioning them as promising candidates for further development as potential LtaS inhibitors.

According to Lu et al. [32], several of these residues have well-defined roles within the LtaS active site: THR300 (here is ALA300 since it is mutated in the used crystal structure 2W5R) is the catalytic residue and a key metal-binding site. GLU255, ASP475, and HIS476 are involved in metal coordination. HIS416 contributes to substrate binding and leaving group protonation. HIS347, ASP349, PHE353, TRY354, ARG356, and LEU384 play roles in substrate binding.

In conclusion, a consistent binding pattern was identified for all hit compounds, including compound **A** and compound **B**, which maintained interactions with key binding residues, particularly GLU255, TRP354, and HIP416. The findings also indicated that while Mn^2+^ serves as a crucial catalytic cofactor in the active site, the inhibition exhibited by the hit compounds does not depend on binding with the Mn^2+^ cation. Instead, the compounds explored interactions with other significant residues in the binding pocket, including GLU255, ALA300, THR352, TRP354, and ARG356.

### 2.4. Molecular Dynamics

**Figure 3:**
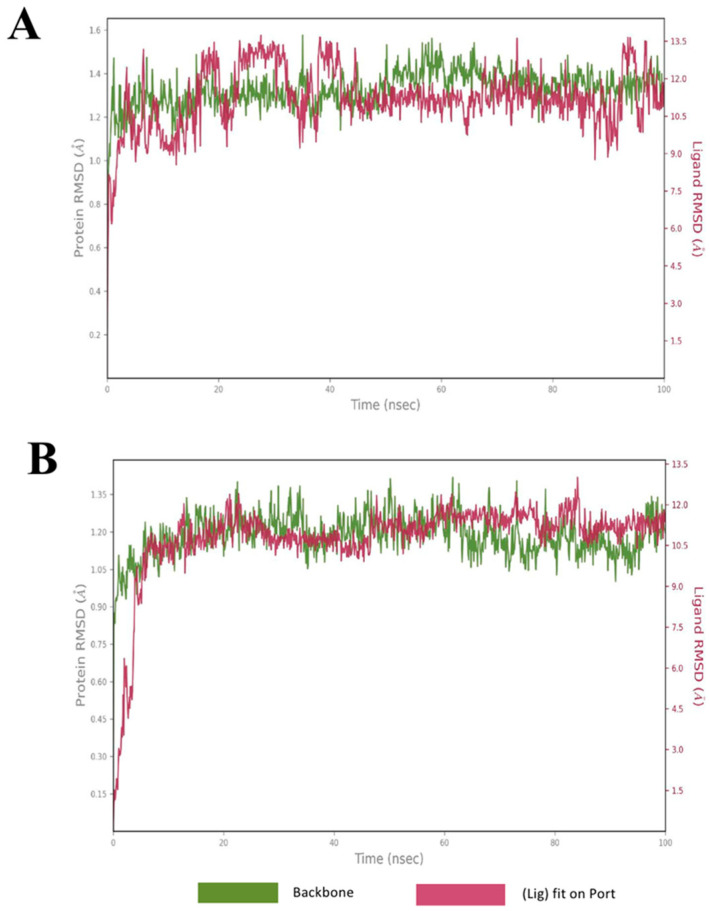
Root mean square deviation (RMSD) graphs for the hit compounds, (**A**): compound **A**, (**B**): compound **B**. The green graph shows fluctuations in the protein backbone from the initial reference point while the red shows the ligand fluctuations. The RMSD profile of the ligand is with respect to its initial fit to the protein binding pocket indicates that all ligands did not fluctuate beyond a 4 Å range after initial stabilization.

**Figure 4:**
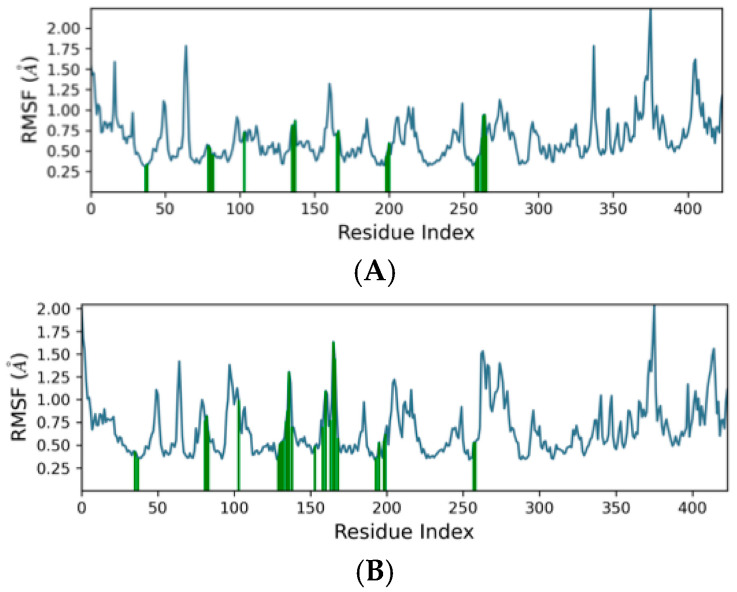
Root mean square fluctuation (RMSF) graphs for the protein complexed with (**A**): Compound **A** and (**B**): Compound **B**. The blue graph shows fluctuations along the protein chain with peaks indicating areas of the protein that fluctuate the most during the simulation. Protein residues that interact with the ligand are marked with green-colored vertical bars.

MD simulations were conducted on the leading compounds identified after virtual screening. Candidate molecules were chosen following a detailed visual inspection of their ligand–protein interaction profiles. Compounds that demonstrated favorable docking scores and strong interactions with critical residues in the binding pocket were advanced to MD studies, which were performed to assess the stability and persistence of binding over a 100-nanosecond trajectory. Among the ligands tested, two displayed the most consistent and stable binding conformations within the binding pocket of the eLtaS protein. To evaluate structural stability, RMSD analysis was applied, measuring the average atomic displacement of each frame relative to a reference structure. The results indicated a stable binding mode, with ligand RMSD fluctuations remaining within approximately 3 Å of the protein backbone throughout the simulation, following an initial equilibration phase of less than 10 nanoseconds. Figure 3 illustrates the RMSD plots for the two top-performing compounds (compound **A** and compound **B**), both of which maintained favorable interactions with key residues in the binding site. Figure 3A reveals that the protein backbone RMSD stabilized between 1.2–1.6 Å after ~20 ns, indicating equilibration and preservation of the global fold. For the first shortlisted compounds (A) the conformational stability of the ligand-protein complex was further supported by residue-level RMSF analysis revealed that fluctuations were concentrated at the N- and C-terminal regions, while structured α-helices and β-strands remained rigid (Figure 4A) when aligned with data from the secondary structure analysis (Figure S1, Supplementary Data). For compound **B** as shown in Figure 3B, the protein backbone RMSD stabilized between 1.0–1.3 Å after ~20 ns. Residue-level RMSF analysis also revealed that fluctuations were concentrated at the N- and C-terminal regions (Figure 4B). For both compounds **A** and **B** loop regions exhibited moderate mobility, as observed in the simulation trajectory, consistent with their role in ligand accommodation. These findings confirm that the protein maintained its native architecture throughout the trajectory, with only localized flexibility.

**Figure 5:**
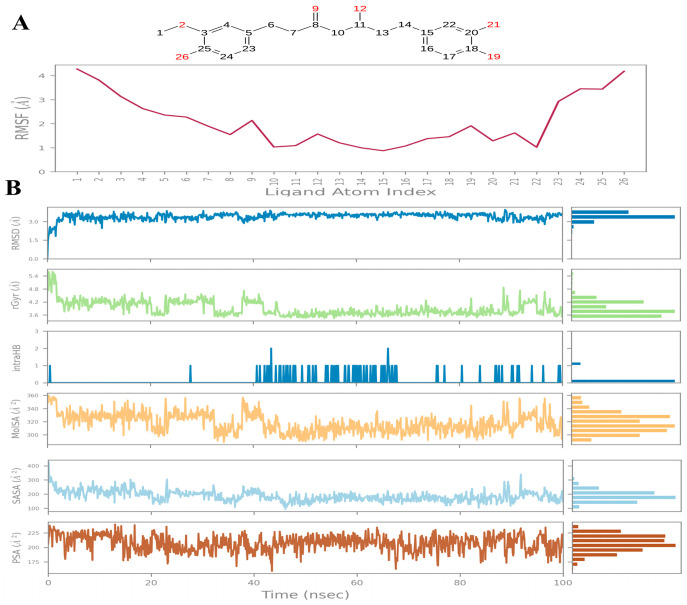
Compound **A**: (**A**) Ligand Root Mean Square Fluctuation. (**B**) Ligand properties: From the top graph: Ligand RMSD: Root mean square deviation of a ligand with respect to the reference conformation; Radius of Gyration (rGyr); Intramolecular Hydrogen Bonds (intraHB): Number of internal hydrogen bonds (HB) within a ligand molecule; Molecular Surface Area (MolSA): Molecular surface calculation with 1.4 Å probe radius; Solvent Accessible Surface Area (SASA); Polar Surface Area (PSA).

For Compound **A** the ligand RMSD fluctuated between 3–5 Å, higher than the protein RMSD, reflecting conformational adjustments within the binding pocket rather than dissociation (Figure 5A). Atom-level RMSF analysis highlighted hydroxyl-bearing substituents as the most mobile, suggesting entropic contributions to binding, as revealed in Figure 5A. Figure 5B describes patterns and fluctuations in detailed ligand properties, including the radius of gyration (Rg) remained between 4.2–4.8 Å, consistent with an extended conformation. Surface property analyses revealed a SASA of 300–400 Å^2^ and PSA of ~200 Å^2^, indicating partial solvent exposure and moderate polarity. The ligand maintained 1–2 intramolecular hydrogen bonds, stabilizing its extended conformation and reducing entropic penalties during binding. Together, these results suggest that the ligand adapts flexibly to the binding pocket while retaining an overall extended scaffold.

**Figure 6:**
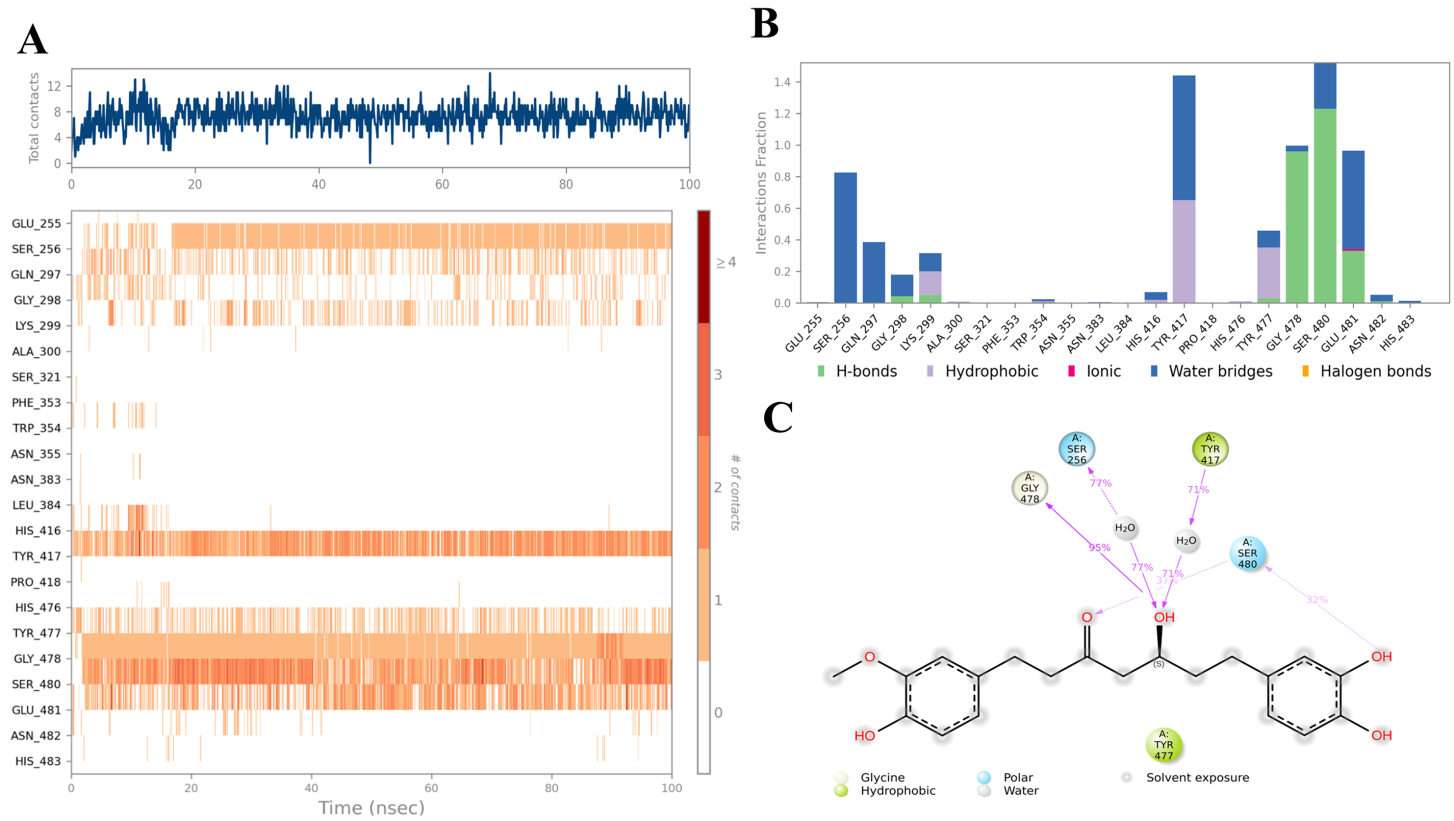
Interaction diagram of hit compound **A** within the eLtaS binding pocket. (**A**) Interaction of compound **A** with residues in each trajectory frame. The depth of color indicating the higher the interaction with contact residues. (**B**) The protein–ligand contacts showing the bonding interactions fraction and the nature of the interactions. (**C**) Graphical 2D illustration of compound **A** interacting with the protein residues during MD simulation.

Compound **A** demonstrated one of the most favorable binding behaviors, engaging with multiple residues within the eLtaS binding pocket, as illustrated in Figure 6. In particular, residues Gly478, Ser480, and Glu481 established stable hydrogen bonds with the ligand. Both Gly478 and Ser480 maintained consistent interactions for more than 60% of the simulation timeframe. Alongside the hydrogen bond and water–bridge interaction formed with Glu481, additional strong water–bridge contacts were observed with Ser256 and Tyr417, persisting for over 70% of the trajectory. Figure 6A,B summarize the key residues that sustained the most stable ligand interactions throughout the entire simulation, while Figure 6C presents all notable contacts between the ligand and binding site residues that occurred for more than 30% of the 100 ns simulation period. Detailed contact analysis revealed persistent interactions with SER256, TYR417, TYR477, GLY478, and SER480, each maintained for >70% of the trajectory. Hydrogen bonds were the dominant stabilizing factor, particularly involving hydroxyl groups of the ligand. Hydrophobic contacts with aromatic residues (PHE353, TRP354, TYR417) reinforced anchoring through π–π stacking. Water-mediated bridges contributed significantly, with bridging contacts present up to 95% of the simulation time, underscoring the role of solvent in stabilizing the complex. The contact timeline demonstrated that the ligand consistently engaged 4–12 residues across the trajectory, with multiple simultaneous contacts ensuring binding persistence.

**Figure 7:**
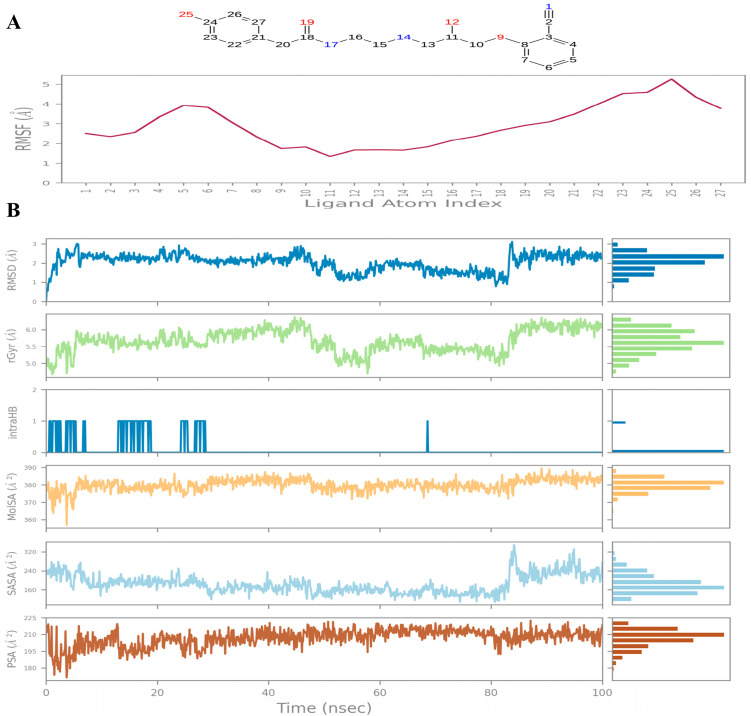
Compound **B**: (**A**) Ligand Root Mean Square Fluctuation. (**B**) Ligand properties: From the top graph: Ligand RMSD: Root mean square deviation of a ligand with respect to the reference conformation; Radius of Gyration (rGyr); Intramolecular Hydrogen Bonds (intraHB): Number of internal hydrogen bonds (HB) within a ligand molecule; Molecular Surface Area (MolSA): Molecular surface calculation with 1.4 Å probe radius; Solvent Accessible Surface Area (SASA); Polar Surface Area (PSA).

For compound **B** ligand RMSD fluctuated between 2–3 Å and atom-level RMSF analysis highlighted flexible substituents, particularly polar and hydroxyl-bearing moieties, which contributed to entropic adaptability during binding (Figure 7A). The radius of gyration (Rg) remained between 5.0–6.0 Å, consistent with an extended conformation. Surface property analyses revealed a SASA of 240–320 Å^2^ and PSA of ~195–225 Å^2^, indicating partial solvent exposure and moderate polarity (Figure 7B). The ligand maintained 1–2 intramolecular hydrogen bonds, stabilizing its extended conformation and reducing entropic penalties during binding.

**Figure 8:**
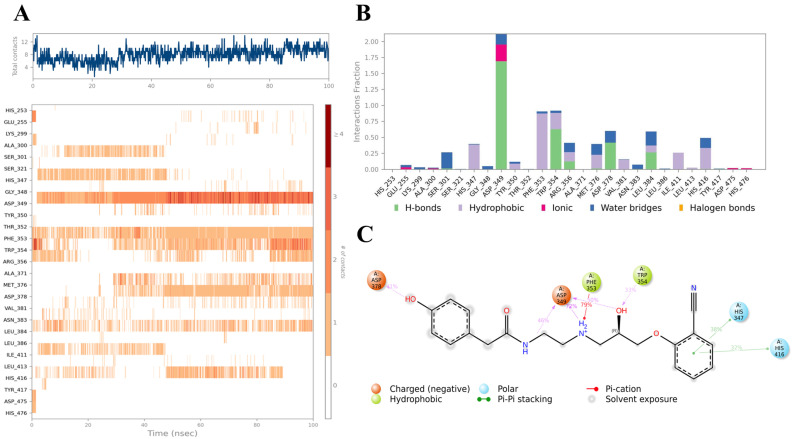
Interaction diagram of hit compound **B** within the eLtaS binding pocket. (**A**) Interaction of compound **B** with residues in each trajectory frame. The depth of color indicating the higher the interaction with contact residues. (**B**) The protein–ligand contacts showing the bonding interactions fraction and the nature of the interactions. (**C**) Graphical 2D illustration of compound **B** interacting with the protein residues during MD simulation.

Compound **B** also displayed a highly favorable binding profile, interacting with several residues within the eLtaS binding pocket, as shown in Figure 8. Notably, Asp349 formed dual hydrogen bonds with both secondary amide groups of the ligand, persisting for approximately 40% and 70% of the simulation time, respectively. An additional ionic interaction at this residue further contributed to the overall stability of the binding fraction throughout the trajectory. A consistent hydrophobic interaction was also observed with Phe353, maintained for nearly 50% of the simulation period. Figure 8A,B highlight the residues that exhibited the most stable ligand interactions across the simulation, while Figure 8C illustrates all significant contacts between the ligand and binding site residues that were sustained for more than 30% of the 100 ns simulation. Detailed contact analysis observed in the simulation trajectory, revealed persistent interactions with SER256, TYR417, TYR477, GLY478, and SER480, each maintained for >70% of the trajectory. Hydrogen bonds were the dominant stabilizing factor, particularly involving hydroxyl groups of the ligand. Hydrophobic contacts with aromatic residues (PHE353, TRP354, TYR417) reinforced anchoring through π–π stacking. Water-mediated bridges contributed significantly, with bridging contacts present up to 95% of the simulation time, underscoring the role of solvent in stabilizing the complex. The contact timeline demonstrated that the ligand consistently engaged 4–12 residues across the trajectory, with multiple simultaneous contacts ensuring binding persistence.

### 2.5. DFT Analysis

**Figure 9:**
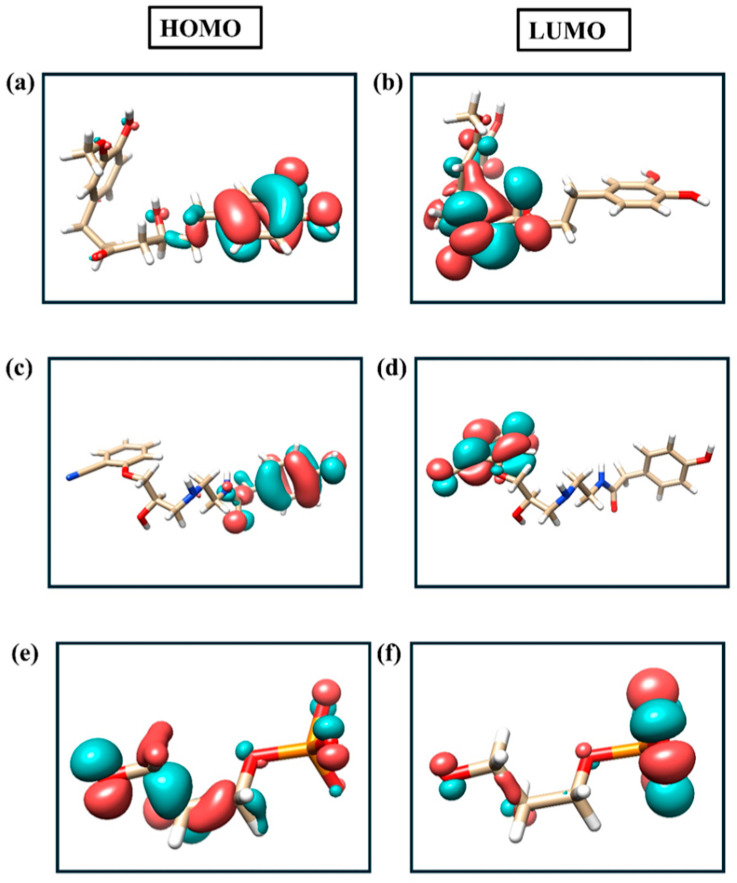
Frontier molecular orbital distributions of the studied compounds. Panels (**a**), (**c**), and (**e**) show the HOMO isosurfaces, while panels (**b**), (**d**), and (**f**) display the corresponding LUMO isosurfaces for CNP0231191.2, CNP0521000.0, and the reference compound.

The electronic structures of the selected compounds and the reference molecule were investigated using DFT calculations, and the corresponding frontier molecular orbitals are illustrated in Figure 9a–f. Panels (a), (c), and (e) depict the HOMO distributions, while panels (b), (d), and (f) represent the corresponding LUMO distributions for compound **A**, compound **B**, and the reference compound, respectively. In both test compounds, the HOMO density is predominantly localized over the conjugated molecular framework, indicating favorable electron-donating capability, whereas the LUMO density extends over adjacent functional moieties, suggesting potential sites for electrophilic interactions. Compared with the reference compound, the spatial separation and delocalization between HOMO and LUMO in the screened compounds indicate improved electronic stabilization and charge-transfer characteristics.

As summarized in Table 2, CNP0231191.2 and CNP0521000.0 exhibit comparable HOMO–LUMO energy gaps of 4.802 eV and 4.665 eV, respectively, which are substantially larger than that of the reference molecule (0.602 eV). The larger energy gaps of the screened compounds imply enhanced kinetic stability and lower intrinsic chemical reactivity relative to the reference. Additionally, both compounds show moderate electronegativity (χ = 0.115852–0.138503 Hartree) and hardness (η = 0.171421–0.176465 Hartree), accompanied by lower electrophilicity indices than the reference, reflecting a balanced donor–acceptor character. Dipole moment analysis further supports these observations. CNP0231191.2 displays a relatively low dipole magnitude (1.534 D), whereas CNP0521000.0 exhibits a higher dipole magnitude (7.615 D), indicative of increased molecular polarity. The reference compound shows an intermediate dipole moment (4.168 D). Collectively, the HOMO–LUMO characteristics, global reactivity descriptors, and dipole moments suggest that the screened compounds possess stable electronic configurations with favorable charge-distribution features when compared to the reference molecule.

### 2.6. ADMET Profiling

The ADMET characteristics of Compounds **A** and **B** were evaluated and compared with a reference compound to assess their drug-likeness and safety profiles (Table 3). Both molecules demonstrated promising oral absorption, with predicted human intestinal absorption values of 76.64% for Compound **A** and 63.67% for Compound **B**, outperforming the reference (57.79%). Although both compounds exhibit relatively low aqueous solubility (log S of −3.711 and −2.798, respectively), they retained acceptable predicted Caco-2 permeability values. Notably, both candidates were identified as P-glycoprotein substrates, indicating a potential for efflux-mediated reduction in intracellular accumulation.

In terms of distribution, both compounds showed negative blood–brain barrier (BBB) permeability (log BB < −0.7) and low CNS penetration values, suggesting limited ability to cross the blood–brain barrier—an advantageous feature for peripheral-acting antibacterial agents. Metabolic profiling indicated distinct liabilities between the two molecules. Compound **A** was predicted to inhibit multiple cytochrome P450 isoforms (CYP1A2, CYP2C19, CYP2C9, and CYP3A4), whereas Compound **B** exhibited a more favorable metabolic profile, being a substrate only for CYP2D6 and not predicted to inhibit major CYP enzymes. Neither compound was predicted to be metabolized by CYP3A4, which may reduce risks of major drug–drug interactions.

Excretion analysis showed moderate predicted total clearance for both molecules, with Compound **B** demonstrating higher clearance compared to Compound **A**. Neither compound was identified as a substrate for renal OCT2 transporters. Importantly, both candidates were predicted to be non-mutagenic (negative AMES test) and non-hERG inhibitors, suggesting a low pro-arrhythmic risk. However, both showed a potential risk for hepatotoxicity, indicating that liver safety should be considered in further optimization and experimental validation.

Collectively, these in silico findings reveal that Compounds **A** and **B** possess favorable oral absorption and safety characteristics but may require structural refinement to mitigate efflux and hepatotoxicity, and to improve solubility and metabolic stability—particularly for Compound **A**, which shows CYP inhibition liabilities.

### 2.7. Physicochemical and Drug-Likeness Profiling

The physicochemical properties of Compounds **A** and **B** were evaluated and benchmarked against a reference molecule to assess their suitability as orally bioavailable drug candidates (Table 4). Both compounds displayed molecular weights within the optimal range for small-molecule therapeutics (360.40 and 370.42 g/mol, respectively), whereas the reference compound exhibited a markedly lower molecular weight (170.06 g/mol). Compound **A** showed moderate lipophilicity (LogP = 2.45), while Compound **B** was more hydrophilic (LogP = 0.53), in contrast to the highly hydrophilic reference (LogP = −1.55). The number of hydrogen-bond donors and acceptors for both compounds remained within accepted drug-likeness thresholds, and their topological polar surface area (TPSA) values (107.22 Å^2^ and 119.19 Å^2^) were consistent with desirable oral bioavailability profiles.

Structural analysis revealed that both hit compounds possess a higher degree of molecular complexity than the reference, as indicated by their increased number of heavy atoms, rotatable bonds, and aromatic centers. Compound **B** had slightly greater flexibility (11 rotatable bonds) than Compound **A** (9 rotatable bonds), while Compound **A** exhibited a marginally higher fraction of sp^3^ carbons, suggesting better three-dimensional character. Both molecules demonstrated high molar refractivity values, supporting favorable molecular volume and polarizability consistent with drug-like behavior.

Drug-likeness filters further supported these observations. Both Compounds **A** and **B** fully satisfied Lipinski, Ghose, Egan, and Muegge criteria, indicating compliance with key physicochemical rules governing oral bioavailability and medicinal chemistry fitness. In contrast, the reference compound triggered multiple violations in the Ghose and Muegge filters due to its smaller size and lower lipophilicity. Bioavailability scores were similar across all molecules (0.55–0.56), suggesting comparable predicted oral availability.

In terms of medicinal chemistry alerts, Compound **A** displayed one PAINS and one Brenk alert associated with the catechol moiety, whereas Compound **B** showed no structural alerts, indicating cleaner chemical space compliance. Synthetic accessibility scores favored the hit compounds (2.94 and 3.23) relative to the reference (3.89), highlighting their feasible synthetic tractability.

Collectively, these results demonstrate that Compounds **A** and **B** possess favorable physicochemical, drug-likeness, and synthetic properties, supporting their potential as viable drug candidates for further biological evaluation. Compound **B** in particular exhibits a more favorable medicinal chemistry profile, with no structural alerts and acceptable scaffold complexity.

## 3. Materials and Methods

### 3.1. Materials and Software

**Figure 10:**
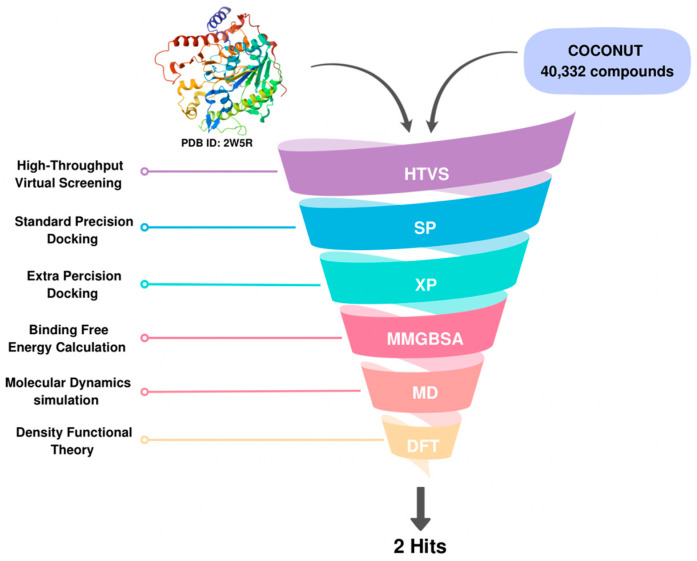
Virtual screening workflow for hit identification. Natural compounds from the COCONUT database (40,332 molecules) were screened against the target protein (PDB ID: 2W5R) using a sequential pipeline: HTVS → SP → XP docking → MM-GBSA → MD simulation. This funnel-based approach yielded two final hit candidates with favorable binding and stability profiles.

The in silico studies were conducted utilizing Schrödinger’s Maestro molecular modeling software version 2024.4 (RDIA RRG Grant 12990-iau-2023-iau-R-3-1-HW: P.O. 6947). The computational work was performed on a desktop workstation equipped with an Intel^®^ Core™ i7-10700F Processor (Intel, Santa Clara, CA, USA), running the Linux Ubuntu 22.10 Operating System, and featuring an RTX 5000 graphics card. The overall work is summarized in Figure 10.

### 3.2. Ligands Retrieval and Preparation

The database of natural products (NPs), comprising 695,133 NPs, was sourced from the Coconut website (https://coconut.naturalproducts.net/, accessed on 20 June 2024). The retrieved structures underwent filtration using Schrödinger’s Canva, based on their physicochemical descriptors, which included molecular weight (MW), logP, number of rings, total heavy atom count, total charge, number of hydrogen donors, number of hydrogen acceptors, number of rotatable bonds, total charge, and quantitative estimate of drug-likeness (QED) score. The final filtration of the database ensured no violations of the Rule of Five and a natural product-like (NPL) score of less than 2 [33]. The ligands were pre-processed using the MacroModel module within the Maestro suite [34]. This preparation step involved optimizing both the geometry and conformational flexibility of the molecules to ensure their suitability for molecular docking. Specifically, energy minimization was performed to refine bond lengths, angles, and torsions, enabling each compound to reach a low-energy, stable conformation. Conformational sampling was also applied to explore various 3D shapes of the ligands and identify possible binding poses. The OPLS force field was employed throughout this process to accurately model the physical and chemical behavior of the compounds.

### 3.3. Protein Retrieval and Preparation

The crystal structure of the extracellular domain of LtaS (eLtaS) with PDB ID: 2W5R was retrieved from the Protein Data Bank (https://www.rcsb.org, accessed on 24 March 2025). The structure was prepared using the Protein Preparation Wizard in Maestro [35]. Initial preprocessing steps included assigning bond orders, adding missing hydrogen atoms, creating zero-order bonds to metals and disulfide bonds, modeling missing side chains and loops, and deleting water molecules beyond 5 Å. Additionally, the Epik tool was used to generate appropriate protonation states at physiological pH (7 ± 2).

Subsequently, the structure was refined by orienting crystallographic water molecules and adjusting the protonation states of residues using PROPKA. Finally, restrained energy minimization was performed using the OPLS4 force field until the heavy atoms converged to a root mean square deviation (RMSD) of 0.30 Å.

### 3.4. Grid Generation and Molecular Docking

Molecular docking was performed using the Glide module in Schrödinger, which offers three levels of precision: high-throughput virtual screening (HTVS), standard precision (SP), and extra precision (XP), each varying in time required and accuracy [26]. A receptor grid was generated from the prepared eLtaS structure using the Receptor Grid Generation tool [27]. The grid box was centered on the crystallographic Mn^2+^ cofactor in the LtaS active site (PDB ID: 2W5R), with outer box dimensions of 20 × 20 × 20 Å and an inner box of 10 Å to encompass surrounding catalytic residues. The Mn^2+^ ion was retained as fixed during grid preparation, with partial charges assigned via the OPLS4 force field and zero-order bonds preserved from protein preparation. The co-crystallized ligand was identified and excluded from the receptor during this step. Default van der Waals scaling parameters were used (scaling factor = 1, partial charge cutoff = 0.25 and no additional positional or hydrogen bonding constraints were imposed to facilitate exploration of metal coordination geometries by ligands.

Docking was carried out via the Ligand Docking panel in Glide. Initially, all 40,332 ligands were docked using HTVS mode. Compounds with the highest docking scores were subsequently re-docked using the SP and then XP modes to refine the binding predictions.

### 3.5. Binding Free Energy Calculations

The MM-GBSA method, implemented via the Prime module, was used to calculate the binding free energies of the top-ranked receptor–ligand complexes [31,36]. The following equation was applied:

∆G = GC − GR − GL

where ∆G is the free binding energy, GC is the target/ligand complex energy, GR is the receptor energy, and GL is the ligand energy. The solvation model was set to be VSGB and the force field was OPLS4.

### 3.6. ADMET Profiling

The pkCSM web server (http://biosig.unimelb.edu.au/pkcsm/prediction, accessed on 2 April 2025) was employed to predict the ADMET parameters, as well as drug-likeness characteristics of the top candidate inhibitors [37]. A total of nineteen molecular descriptors were utilized to evaluate the ADMET profiles of the proposed LtaS hits. Furthermore, the SwissADME platform (www.swissadme.ch, accessed on 2 April 2025) was used to determine the physicochemical properties, medicinal chemistry features, and drug-likeness parameters of the compounds [38]

### 3.7. Molecular Dynamics (MD) Simulations

Top-performing compounds based on MM-GBSA scores were subjected to molecular dynamics simulations [39,40]. In summary, the ligand–protein complex, obtained in its most favorable docking pose, was first energy-minimized using the Protein Preparation Wizard and then set up for simulation through Desmond’s system builder. The simulation system employed a TIP3P water model as the solvent. An orthorhombic box was constructed with a 10 Å buffer surrounding the protein, and the system was neutralized by introducing the appropriate counterions along with 0.15 M NaCl to replicate physiological salt concentration. The MD run was conducted under standard conditions of 300 K and 1.013 bar. Standard settings were used for selected relaxation protocols including a thermostat method: Nose-Hoover chain at a relaxation interval of 1 picosecond (ps) and a barostat method of Martyna-Tobias-Klein at a relaxation time of 2 ps with an isotropic coupling style. Each trajectory was generated over a 100-nanosecond timescale, with more than 1000 frames recorded for subsequent analysis. Post-simulation evaluation and visualization were carried out using Desmond’s Simulation Interaction Diagram tool.

### 3.8. Density Functional Theory (DFT) Calculations

The optimized geometry of the ligand was subjected to quantum mechanical calculations using the PySCF framework [41]. Molecular preparation, including hydrogen addition and geometry optimization, had been carried out using RDKit (https://www.rdkit.org/, accessed on 30 November 2025) in advance of quantum mechanical calculations. In this study, DFT calculations were conducted using an exchange-correlation functional, B3LYP, in combination with the cc-pVDZ basis set [42,43,44]. Frontier molecular orbitals (HOMO and LUMO) are generated as cube files to view the HOMO and LUMO distributions, whereas total energy, HOMO-LUMO energy gaps, dipole moment, Mulliken atomic charges, and some conceptual DFT reactivity descriptors such as electronegativity, hardness, softness, and electrophilicity index were calculated to characterize the electronic stability and reactivity of the ligand.

## 4. Conclusions

Our research study successfully demonstrates the application of an integrated computational strategy to identify potential inhibitors of the *S. aureus* LtaS enzyme. By employing a sequential workflow of multi-stage molecular docking (HTVS, SP, then XP), we efficiently narrowed down a vast natural compound library from the COCONUT database to a manageable number of high-affinity candidates. The subsequent application of MM-GBSA binding free energy calculations provided a more robust estimate of ligand-binding affinity, serving as a critical filter to prioritize the most promising hits. In addition, the 100 ns molecular dynamics simulations offered profound insights into the stability and the dynamics of the protein–ligand complexes. The results confirmed that the top candidates, particularly Compound **A** and compound **B**, formed stable interactions with the active site of eLtaS, maintaining low RMSD and favorable interaction profiles throughout the simulation period. This indicates their potential to effectively inhibit the enzyme’s function under physiological conditions. The Density Functional Theory findings also underscore the enhanced electronic stability and charge-transfer capabilities of the screened compounds, providing crucial insights for their optimized design and potential application in future lead generation. Furthermore, the comprehensive ADMET profiling confirmed that these lead compounds possess desirable pharmacokinetic properties and a low probability of toxicity, aligning with the prerequisites for a viable drug candidate. In summary, this study not only validates eLtaS as a druggable target but also shortlists eight structurally diverse initial hits and identifies Compound **A** and compound **B** as highly promising, naturally derived scaffolds for inhibiting its activity. These findings provide a strong rationale for subsequent in vitro and in vivo experimental studies to confirm their efficacy against *S. aureus*, setting the ground for a new class of anti-staphylococcal therapeutics.

## Figures and Tables

**Table 1:** Docking scores and MM-GBSA ΔG_bind energies of the top compounds and the natural substrate (reference).

Compound	COCONUT ID	Chemical Structure	Docking Score in kcal/mol	ΔG_bind in kcal/mol	Key Amino Acid Residues in LtaS	Interactions
**A**	CNP0231191.2	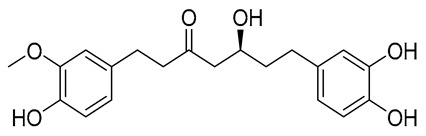	−6.771	−46.02	HIP416, THR300, GLU255, TRP354, ASP475, ASP349, ARG356, LEU384 and HIP476	THR352, TYR417 H_2_O2002, and 2435
**B**	CNP0521000.0	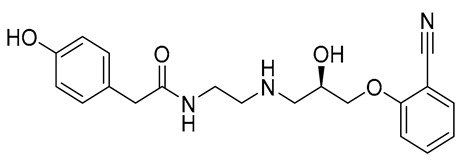	−6.414	−32.78		THR352, TRP354, HIP416, and H_2_O2002
**C**	CNP0471911.0	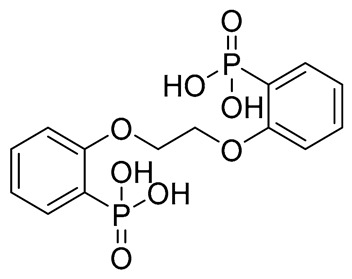	−11.285	−26.32		GLU255, PHE353, TRP354, HIP416, and H_2_O2142
**D**	CNP0470462.0	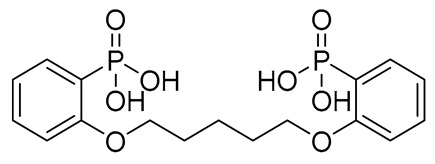	−11.27	−20.3		GLU255, TRP354, HIP416, and H_2_O2142
**E**	CNP0598632.1	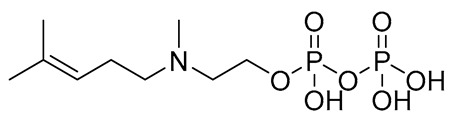	−11.169	−5.72		GLU255, ALA300, ARG356, HIP416, and H_2_O2142
**F**	CNP0471223.0	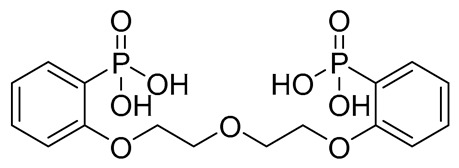	−11.012	−22.56		GLU255, TRP354, ASN383, HIP416, TYR417, and H_2_O2142
**G**	CNP0470600.0	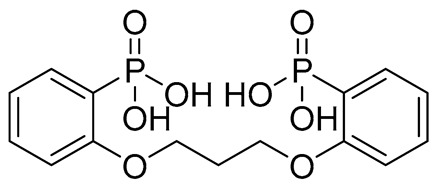	−10.912	−19.56		GLU255, TRP354, HIP416, and H_2_O2142
**H**	CNP0477679.0	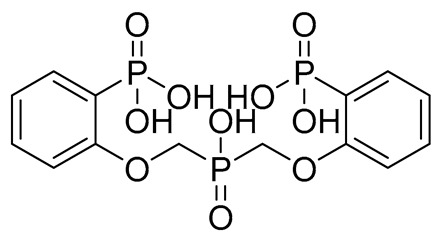	−10.895	−3.37		GLU255, TRP354, HIP416, and H_2_O2142
Reference		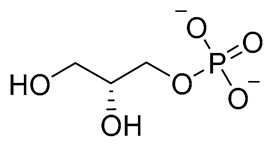	−10.062	−30.15		ALA300, TRP354, ARG356, HIS347 MN1643, H_2_O2440, and 2441

**Table 2:** Density functional theory-derived electronic properties of the selected compounds and the reference molecule, including total energy, HOMO and LUMO energies, HOMO–LUMO energy gap, dipole moment, and global conceptual DFT reactivity descriptors.

S. No.	Compounds	Total Energy (Hartree)	HOMO Energy (Hartree)	LUMO Energy (Hartree)	HOMO-LUMO Gap	Electronegativity (χ) (Hartree)	Hardness (η) (Hartree)	Softness (S)	Electrophilicity Index (ω) (Hartree)
1.	**A**	−1227.931989	−0.204084	−0.027620	4.802 eV	0.115852	0.176465	5.666852	0.038029
2.	**B**	−1241.137531	−0.224214	−0.052793	4.665 eV	0.138503	0.171421	5.833580	0.055953
3.	Reference	−911.096591	−0.288515	−0.266392	0.602 eV	0.277454	0.022123	45.200991	1.739797

**Table 3:** Predicted ADMET and Permeability Parameters for Hit Compounds **A** and **B**.

ADMET Parameters	Compound A	Compound B	Reference
**Absorption**			
Water solubility (log mol/L)	−3.711	−2.798	−0.02
Caco2 permeability (log Papp in 10^−6^ cm/s)	0.156	0.069	−0.193
Intestinal absorption (human) (% Absorbed)	76.64	63.667	57.786
P-glycoprotein substrate (Yes/No)	Yes	Yes	No
**Distribution**			
BBB permeability (log BB)	−1.033	−0.79	−0.896
CNS permeability (log PS)	−3.329	−3.608	−3.67
**Metabolism**			
CYP2D6 substrate (Yes/No)	No	Yes	No
CYP3A4 substrate (Yes/No)	No	No	No
CYP1A2 inhibitor (Yes/No)	Yes	No	No
CYP2C19 inhibitor (Yes/No)	Yes	No	No
CYP2C9 inhibitor (Yes/No)	Yes	No	No
CYP2D6 inhibitor (Yes/No)	No	No	No
CYP3A4 inhibitor (Yes/No)	Yes	No	No
**Excretion**			
Total Clearance (log mL/min/kg)	0.299	1.164	0.34
Renal OCT2 substrate (Yes/No)	No	No	No
**Toxicity**			
AMES toxicity (Yes/No)	No	No	No
Max. tolerated dose (human) (log mg/kg/day)	0.158	−0.273	1.439
hERG I inhibitor (Yes/No)	No	No	No
Hepatotoxicity (Yes/No)	Yes	Yes	No

**Table 4:** Predicted Physicochemical properties, drug-likeness, and medicinal chemistry for Hit Compounds **A** and **B**.

Molecule Properties	Compound A	Compound B	Reference
**Physicochemical properties**			
Molecular Weight	360.40 g/mol	370.42 g/mol	170.06 g/mol
LogP	2.45	0.53	−1.55
#Acceptors	6	5	6
#Donors	4	4	2
#Heavy atoms	26	27	10
#Arom. heavy atoms	12	12	0
Fraction Csp3	0.35	0.30	1
#Rotatable bonds	9	11	4
Molar refractivity	98.66	101.22	27.87
TPSA (Å^2^)	107.22	119.19	122.69
**Drug-likeness**			
Lipinski alert	Yes; 0 violation	Yes; 0 violation	Yes; 0 violation
Ghose	Yes	Yes	No; 3 violations: WLOGP < −0.4, MR < 40, #atoms < 20
Veber	Yes	No; 0 violation: Rotors > 10	Yes
Egan	Yes	Yes	Yes
Muegge	Yes	Yes	No; 3 violations: MW < 200, XLOGP3 < −2, #C < 5
Bioavailability Score	0.55	0.55	0.56
**Medicinal chemistry**			
PAINS	1 alert: catechol_A	0 alert	0 alert
Brenk	1 alert: catechol	0 alert	1 alert: phosphor
Synthetic accessibility	2.94	3.23	3.89

## Data Availability

The data used in this study are public, and analyzed data are in the manuscript as figures and tables. Further inquiries can be directed to the corresponding authors.

## Supplementary Materials

The supporting information can be downloaded at: https://www.mdpi.com/article/10.3390/ijms262412038/s1.
