# Supplementary material for: In Silico Lead Identification of Staphylococcus aureus LtaS Inhibitors: A High-Throughput Computational Pipeline Towards Prototype Development
Source: Int J Mol Sci. 2025 Dec 14;26(24):12038. doi: 10.3390/ijms262412038 (PMC12732334; doi:10.3390/ijms262412038)
Supplement: Supplementary file 1 [file ijms-26-12038-s001.zip › ijms-4018366-supplementary.pdf]

Supplementary figures:

Figure S1:

A

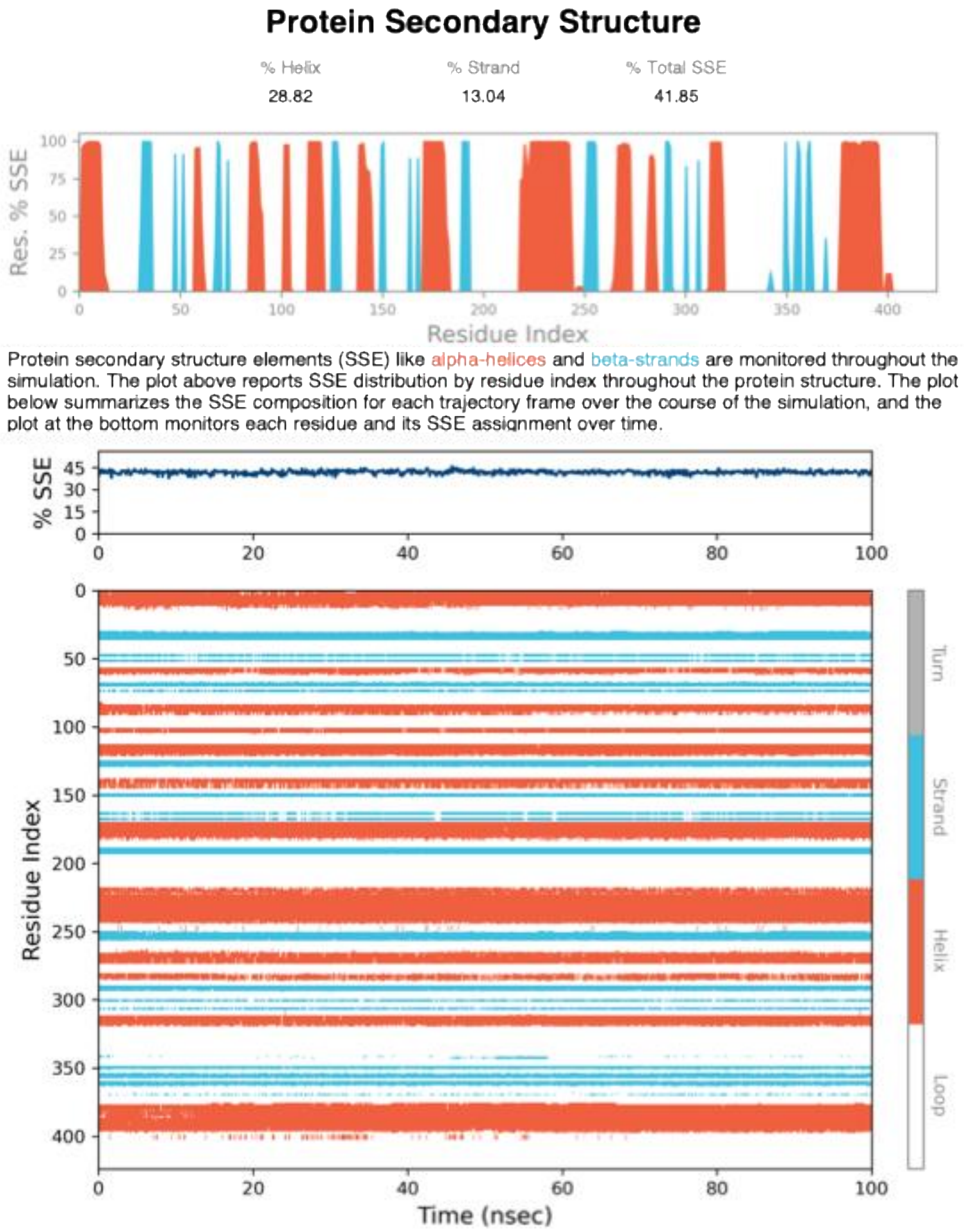

B

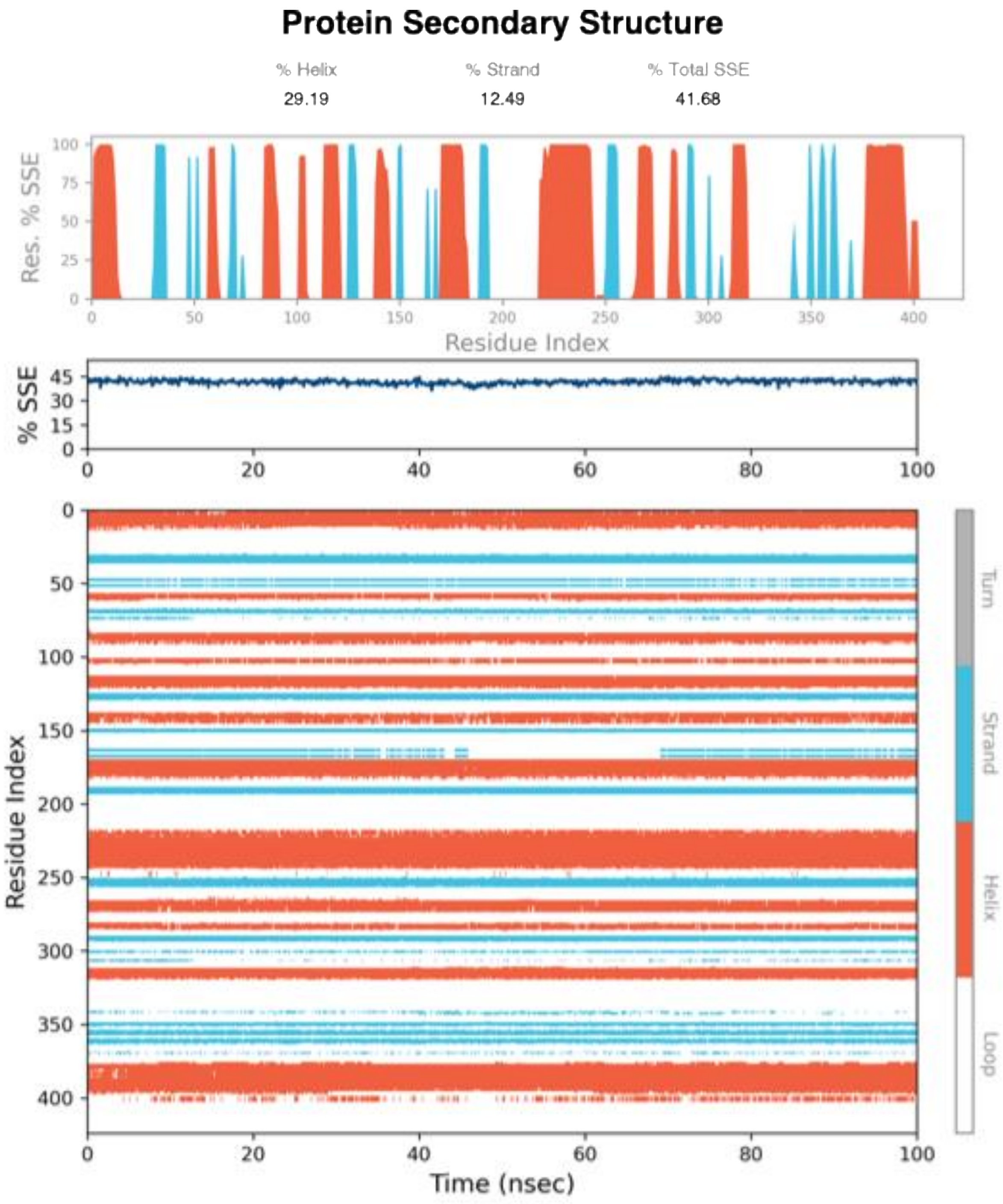

Protein secondary structure analysis for (A) **Compound A** (B) **Compound B**: secondary structure elements (SSE) like alpha-helices (orange) and beta-strands (blue) are monitored throughout the simulation. The plot above reports SSE distribution by residue index throughout the protein structure. The middle plot summarizes the SSE composition for each trajectory frame over the course of the simulation, and the plot at the bottom monitors each residue and its SSE assignment over time.
